# Retrotransposons as a Source of DNA Damage in Neurodegeneration

**DOI:** 10.3389/fnagi.2021.786897

**Published:** 2022-01-04

**Authors:** Eugenie Peze-Heidsieck, Tom Bonnifet, Rania Znaidi, Camille Ravel-Godreuil, Olivia Massiani-Beaudoin, Rajiv L. Joshi, Julia Fuchs

**Affiliations:** Center for Interdisciplinary Research in Biology (CIRB), CNRS, INSERM, Collège de France, Université PSL, Paris, France

**Keywords:** transposable elements (TEs), genomic instability, LINE-1, DNA damage, neurodegenerative diseases, Parkinson’s disease, Alzheimer’s disease, aging

## Abstract

The etiology of aging-associated neurodegenerative diseases (NDs), such as Parkinson’s disease (PD) and Alzheimer’s disease (AD), still remains elusive and no curative treatment is available. Age is the major risk factor for PD and AD, but the molecular link between aging and neurodegeneration is not fully understood. Aging is defined by several hallmarks, some of which partially overlap with pathways implicated in NDs. Recent evidence suggests that aging-associated epigenetic alterations can lead to the derepression of the LINE-1 (Long Interspersed Element-1) family of transposable elements (TEs) and that this derepression might have important implications in the pathogenesis of NDs. Almost half of the human DNA is composed of repetitive sequences derived from TEs and TE mobility participated in shaping the mammalian genomes during evolution. Although most TEs are mutated and no longer mobile, more than 100 LINE-1 elements have retained their full coding potential in humans and are thus retrotransposition competent. Uncontrolled activation of TEs has now been reported in various models of neurodegeneration and in diseased human brain tissues. We will discuss in this review the potential contribution of LINE-1 elements in inducing DNA damage and genomic instability, which are emerging pathological features in NDs. TEs might represent an important molecular link between aging and neurodegeneration, and a potential target for urgently needed novel therapeutic disease-modifying interventions.

## Introduction

Age-associated neurodegenerative diseases (NDs) such as Parkinson’s disease (PD) and Alzheimer’s disease (AD) have become a global burden due to the continued increase in life expectancy with obvious socio-economic implications (Yang et al., 2020). The support and care of people with NDs, for which age is the main known risk factor, poses a major challenge. Unfortunately, currently available treatments only alleviate some of the symptoms and there is still no disease-modifying treatment (Van Bulck et al., 2019).

Neurodegenerative diseases are clinically separated into specific syndromes based on typical clinical manifestations. For instance, the loss of dopaminergic neurons in the *substantia nigra pars compacta* (SNpc) leads to the cardinal motor symptoms in PD (Kalia and Lang, 2015), whereas the degeneration of hippocampal and cortical neurons results in memory impairment, cognitive dysfunction, and dementia in AD (Oboudiyat et al., 2013). In spite of these clinical differences, NDs present substantial neuropathological and genetic overlap (Naz et al., 2017; Gan et al., 2018; Karch et al., 2018). Pathways altered in various NDs include protein quality control, the autosomal-lysosome pathway, mitochondrial homeostasis, protein seeding, propagation of stress granules, synaptic toxicity, and network dysfunction (Gan et al., 2018). Among genetic and environmental factors, age remains the major risk factor for the development of the most prevalent NDs like PD and AD, as well as for other NDs like amyotrophic lateral sclerosis (ALS), multisystem atrophy (MSA), Lewy body disease (LBD), frontotemporal dementia (FTD), and Huntington’s disease (HD) (Kritsilis et al., 2018; Hou et al., 2019).

This review will highlight some striking similarities between the aging process and known pathways involved in the pathogenesis of NDs, discuss how the failure of multiple layers of LINE-1 repression, related to the pathogenesis of NDs, could explain an age-related derepression of TEs and particularly focus on LINE-1 as a source of genomic instability, an emerging pathway triggering neurodegeneration. We will also discuss a hypothetical role of LTR (long terminal repeat) retrotransposons as a possible additional source of DNA damage in the brain. Many other pathogenic mechanisms through which TEs might act as pathogenic drivers in human diseases including neurodegenerative diseases have been extensively reviewed elsewhere (Gorbunova et al., 2021; Ravel-Godreuil et al., 2021b).

## Parallels Between Neurodegenerative Diseases and the Aging Process

To date, despite enormous efforts and a tremendous increase in knowledge about the fundamental nature of NDs, it remains widely unknown how aging and NDs might be linked at the molecular level. Aging is defined as a progressive loss of physiological integrity, leading to impaired functions and increased vulnerability to death (Gilbert, 2000; López-Otín et al., 2013). Aging, as defined by the “disposable theory of aging” put forward by Thomas Kirkwood in 1977, is due to a gradual, life-long accumulation of faults (e.g., DNA damage) in human cells and tissues, leading to organ dysfunction, disease, and ultimately death. So far, nine hallmarks of aging have been defined which comprise genomic instability, telomere attrition, epigenetic alterations, loss of proteostasis, deregulated nutrient sensing, mitochondrial dysfunction, cellular senescence, stem cell exhaustion, and altered intracellular communication (López-Otín et al., 2013). The first four hallmarks are considered causative primary hallmarks, which initiate cellular damage. Since age is the major risk factor for NDs, it is important to understand whether and how aging-related processes participate in the pathogenesis of NDs. Table 1 presents a compilation of existing evidence that links NDs with what has been defined as hallmarks of organismal aging. Evidence for the presence of all the nine aging hallmarks has been documented either in brain aging or in the context of NDs (Mattson and Arumugam, 2018; Hou et al., 2019), but we will highlight here the two primary aging hallmarks, namely, genomic instability and epigenetic alterations, as these are most relevant to TE biology.

**TABLE 1:** Evidence for aging hallmarks in the healthy or diseased brain.

The nine hallmarks of aging The hallmarks of aging (López-Otín et al., 2013)	Evidence for aging hallmarks in the healthy or diseased brain
Genomic instability	DNA damage and its links to neurodegeneration (Madabhushi et al., 2014) Engrailed homeoprotein blocks degeneration in adult dopaminergic neurons through LINE-1 repression (Blaudin de Thé et al., 2018) Deletion of topoisomerase 1 in excitatory neurons causes genomic instability and early onset neurodegeneration (Fragola et al., 2020) Inefficient DNA repair is an aging-related modifier of Parkinson’s Disease (Sepe et al., 2016)
Telomere attrition	Debated: Eitan et al. (2014) Telomere shortening in neurological disorders: an abundance of unanswered questions Folic acid inhibits aging-induced telomere attrition and apoptosis in astrocytes *in vivo* and *in vitro* (Li et al., 2021)
Epigenetic alterations	Epigenetic regulation in neurodegenerative diseases (Berson et al., 2018) Epigenetic changes and its intervention in age-related neurodegenerative diseases (Mohd Murshid et al., 2020)
Loss of proteostasis	Regulation of protein homeostasis in neurodegenerative diseases: the role of coding and non-coding genes (Sin and Nollen, 2015) Altered proteostasis in neurodegenerative tauopathies (Papanikolopoulou and Skoulakis, 2020) Proteostasis disturbances and inflammation in neurodegenerative diseases (Sonninen et al., 2020)
Altered intercellular communication	On the central role of brain connectivity in neurodegenerative disease progression (Iturria-Medina and Evans, 2015) Extracellular vesicles and neurodegenerative diseases (Hill, 2019)
Stem cell exhaustion	Nutrients, neurogenesis and brain aging: From disease mechanisms to therapeutic opportunities (Fidaleo et al., 2017) Stem cell aging in lifespan and disease: A state-of-the-art review (Sameri et al., 2020)
Cellular senescence	Aging, cellular senescence and neurodegenerative disease (Kritsilis et al., 2018) Cellular senescence in brain aging and neurodegenerative diseases: evidence and perspectives (Baker and Petersen, 2018)
Mitochondrial dysfunction	Abnormalities of mitochondrial dynamics in neurodegenerative diseases (Gao et al., 2017) Mitochondrial dysfunction in neurodegenerative diseases and the potential countermeasure (Wang et al., 2019) Mitochondrial dysfunction in the development and progression of neurodegenerative diseases (Johnson et al., 2021)
Deregulated nutrient sensing	Dysregulation of nutrient sensing and CLEARance in presenilin deficiency (Reddy et al., 2016)

## Neurodegeneration, Aging, and Genomic Instability

It is well documented that aging cells accumulate persistent DNA damage throughout life (Sedelnikova et al., 2004) and DNA repair activity declines in neurons with aging (Lu et al., 2004; Vyjayanti and Rao, 2006). Post-mitotic neurons in the central nervous system are particularly susceptible to this type of damage. This is due mainly to two factors related to the specificities of neuronal cell functioning. First, post-mitotic neurons do not dispose of the full repertoire of DNA repair pathways to repair DNA double-strand breaks (DSBs) (Chow and Herrup, 2015). Indeed, in mammals, there are four main DNA repair pathways, namely, nucleotide excision repair (NER) and base excision repair (BER) to repair single-strand lesions and base alterations, respectively, and homologous recombination (HR) and non-homologous end-joining (NHEJ) to repair DSBs (Chatterjee and Walker, 2017). HR requires DNA replication during cell division. However, neurons are post-mitotic, hence non-dividing and therefore cannot rely on the HR repair pathway but instead exclusively depend on NHEJ known to be more error prone. The second main specificity of neurons is the high metabolic rate which renders them prone to metabolic stress, resulting in elevated oxidative stress through the production of free radicals leading to DNA damage (Ismail and Hendzel, 2008). DNA damage can also be exacerbated by the defective functioning of topoisomerases in the context of NDs. In a physiological context, topoisomerases release torsional stress during DNA transcription, particularly on extremely long genes enriched in neuronal functions (King et al., 2013) and coordinate transcription from the promoter of immediate early genes associated with learning and memory (Madabhushi et al., 2015). These studies suggest that DNA strand breaks, if rapidly repaired at steady-state levels, can participate in physiological neuronal functions. However, when DNA repair becomes altered during aging, the transcription-associated and topoisomerase-linked DNA damage can accumulate and have indeed been reported as elevated in the context of AD (Suberbielle et al., 2013). This illustrates the importance of endogenous sources of genomic instability. The particular vulnerability of neurons to DNA damage is underscored by the fact that numerous diseases, linked to mutations in DNA repair factors, manifest with neurological symptoms (Katyal et al., 2014; Madabhushi et al., 2014). Furthermore, each neuronal population, or even subpopulation, carries cell-type-specific vulnerabilities. For instance, dopaminergic neurons are particularly vulnerable to oxidative stress due to their specific physiology and morphology (Surmeier et al., 2017), especially in humans where dopaminergic neurons increased in number and arborization throughout evolution (Vernier, 2004; Bolam and Pissadaki, 2012).

DNA damage has been documented in experimental models of NDs, and in PD and AD patients (Mullaart et al., 1990; Adamec et al., 1999; Sepe et al., 2016; Mitra et al., 2019; Shanbhag et al., 2019). Interestingly, mice deficient in the serine/threonine kinase ataxia telangiectasia (ATM) show a selective degeneration of dopaminergic neurons in the SNpc (Eilam et al., 1998). Following rapid recruitment to DSBs, ATM not only coordinates several aspects of the cellular DNA damage response (Shiloh and Ziv, 2013) but also averts DNA damage by preventing the accumulation of topoisomerase-dependent DNA lesions (Katyal et al., 2014). This indicates that dopaminergic neurons are particularly sensitive to defects in the DSB repair pathway. The NER pathway is equally important in dopaminergic neurons as NER-deficiency induces a PD-related pathology (Sepe et al., 2016). More recently, DSBs were shown to precede all pathological hallmarks in a mouse model of AD (CK-p25). DNA damage in these mice coincided with a reduction in HDAC1 (histone deacetylase 1), and neuronal loss could be rescued by HDAC1 (Kim et al., 2008) and the NAD^+^-dependent deacetylase SIRT1 (Sirtuin 1) (Kim et al., 2007). A complex interplay between HDAC1, SIRT1, and ATM was identified, suggesting that unrepaired DSBs, due to a dysfunctional DNA repair pathway, could underline neurodegeneration in AD (Dobbin et al., 2013). DNA damage has also been shown to accumulate in other NDs such as ALS (Kwiatkowski et al., 2009; Vance et al., 2009; Wang et al., 2013).

Taken together, neurons seem particularly susceptible to DNA damage due to neuron-specific features. Increasing experimental evidence suggests that genomic instability is related to the aging process in neurons and sufficient to trigger neurodegeneration. The life-long accumulation of DNA damage together with a decline in DNA repair mechanisms might be initiating or at least contributing to neurodegeneration (Madabhushi et al., 2014; Martínez-Cué and Rueda, 2020).

We discuss below the emerging concept that LINE-1 elements might be an additional source of DNA damage and genomic instability and that LINE-1 activation in the brain could be part of the aging process as shown in somatic cells (Gasior et al., 2006; Belancio et al., 2010; De Cecco et al., 2019; Simon et al., 2019) and lead to DNA damage and neurodegeneration (Blaudin de Thé et al., 2018). Through induction of genomic instability and other recently discovered consequences of LINE-1 activation, e.g., neuroinflammation (reviewed in Saleh et al., 2019; Gorbunova et al., 2021), LINE-1 represents a so far unsuspected new pathogenic driver in NDs.

## Life Cycle of Line-1 Retrotransposons

**FIGURE 1:**
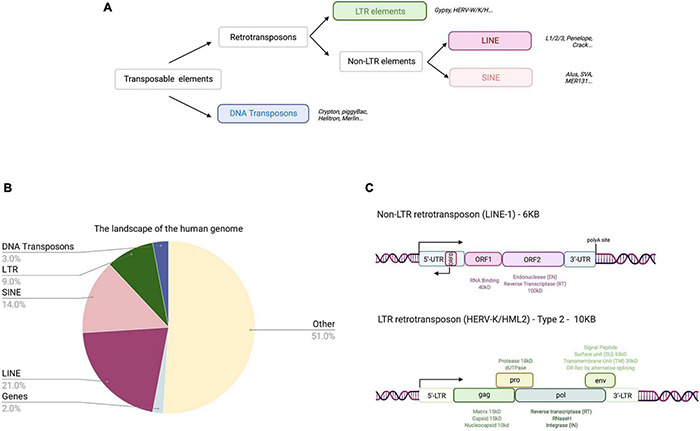
Categories of TEs in the human genome and their coding potential. **(A)** TE classification. TEs in humans can be classified into four categories belonging to two classes, retrotransposons (class 1) and DNA transposons (class 2). Retrotransposons include non-LTR retrotransposons (autonomous LINEs and non-autonomous SINEs) and LTR retrotransposons. Examples of elements are written in italics and boxed families are represented in the pie chart (**B**) indicating relative% abundance in the human genome. **(B)** The landscape of the human genome. The human genome consists of sequences derived from TEs (47%), coding sequences (or exons; 2%), and “other” sequences (promoters, enhancers, introns, non-coding RNA, telomeres, centromeres, and pseudogenes; 51%). **(C)** Human retrotransposons with coding potential. Non-LTR retrotransposons (approximately 6kB long) are composed of a 5′UTR (containing sense and antisense promoters), two open reading frames (ORF1 and ORF2), and a 3′UTR containing a poly(A) site. ORF1 encodes for an RNA binding protein and ORF2p has EN and RT activities. Human and primate LINE-1 also encode an antisense ORF, termed ORF0 present in the 5′UTR. LTR (HERV-K/HML-2) retrotransposons (approximately 10 kB long) are flanked by two LTRs. They contain a 5′UTR promoter within the LTR, a primer binding site and four main ORFs (gag, pro, pol, and env) giving rise to Gag, Gag-Pro-Pol, and Gag-Pro polypeptides (*via* ribosomal frameshift) and Env. Gag is cleaved by the encoded viral protease into the matrix, capsid and nucleocapsid proteins, which have structural functions. The protease is autocleaved into the viral protease and a dUTPase. Gag-Pol polyprotein cleavage by the viral protease gives rise to RT with polymerase activities, RNaseH, and IN. The Env protein is generated from a spliced mRNA and cleaved in the endoplasmatic reticulum by a cellular protease into signal peptide, surface unit, and transmembrane unit. Alternative splicing of Env generates two other proteins depending on the HERV-K/HML-2 type: rec or Env (type 2) or np9, but no Env (type 1, *not shown*).

**FIGURE 2:**
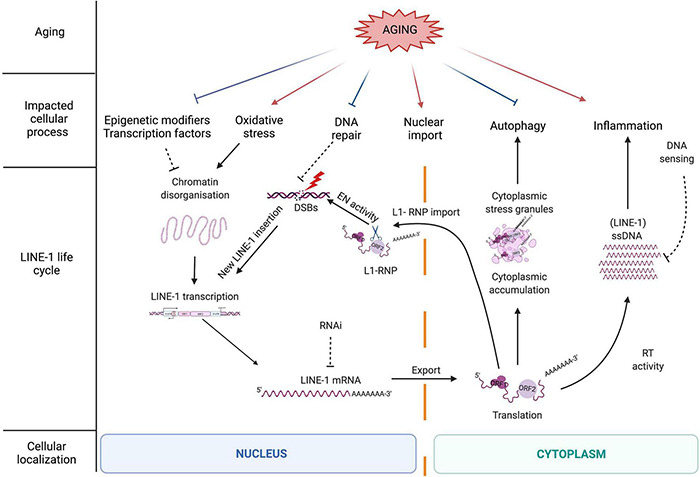
The LINE-1 retrotransposition life cycle as a source of DNA damage and the influence of the aging process on LINE-1 repressive mechanisms. The LINE-1 life cycle is controlled by different cellular processes many of which are negatively impacted by aging (described in more detail in the main text). Aging might thus contribute to a release of repression of LINE-1 by cellular control mechanisms including epigenetic repression, DNA repair, and autophagy. On the other hand, other cellular processes impacted by aging favor either LINE-1 expression (oxidative stress) or possibly LINE-1 nuclear import (increase in nuclear pore permeability). Aging-related inflammation might be amplified by LINE-1 expression through the generation of cytoplasmic nucleic acids via the LINE-1 RT and the generation of ssDNA which is repressed by DNA sensing (i.e., via the exonuclease Trex1 or other proteins involved in the modulation of cytosolic nucleic acid species). When cellular repressive mechanisms are alleviated, full-length LINE-1 elements become expressed, which initiates the LINE-1 life cycle starting with the transcription of a full-length LINE-1 element from the endogenous promoter contained in the 5′UTR (Figure 1C) and the export of the polyA + LINE-1 mRNA into the cytoplasm. Once translated, the LINE-1 encoded proteins ORF1p and ORF2p reassemble in “cis” with the LINE-1 mRNA to form a ribonucleoparticle (RNP). The LINE-1 RNP can accumulate in the cytoplasm in stress granules. Through a widely unknown mechanism, the RNP enters the nucleus where the ORF2 EN creates DNA strand breaks and a new LINE-1 copy (often 5′truncated) is reverse transcribed into the genome *via* target-primed reverse transcription (TPRT). ORF2p can also create DNA strand breaks independent of retrotransposition. As a bystander of cytoplasmic RT activity and *via* an unknown primer, ORF2 RT can reverse transcribe RNA into ssDNA which triggers the innate immune system and inflammation.

The complete human genome sequencing revealed that about 50% of human DNA consists of repetitive sequences (Lander et al., 2001), most of which are remnants of an ancient activity of TEs (Figures 1A,B and Table 2). These mobile elements comprise DNA transposons, LTR-retrotransposons (mammalian apparent LTR retrotransposon, MaLRs; endogenous retroviruses, ERVs), and non-LTR retrotransposons (LINE-1; Short INterspersed Elements, SINEs, and the composite element SINE/VNTR/Alu, SVA). LINE-1 elements have massively expanded in mammalian genomes and are the only autonomous retrotransposons in humans that encode their own mobilization machinery to move from one genomic location to another. As shown in Figure 1C, a full-length LINE-1 contains two open reading frames: ORF1 coding for ORF1p (an RNA binding protein) and ORF2 encoding ORF2p (with endonuclease, EN, and reverse transcriptase, RT, activity). The number of LINE-1 elements that have retained their full coding potential in the human genome is currently estimated to be 146 in the human reference genome GRCh38/hg38 and 2811 in the mouse reference genome GRCm38/mm10 (euL1db: L1Basev2 Mir et al., 2015; Penzkofer et al., 2017). The mechanism of LINE-1 retrotransposition, the “LINE-1 life cycle,” is depicted in Figure 2 and described in more detail in the figure legend. TEs have self-amplified and shaped mammalian genomes during evolution and possibly conferred evolutionary benefit (Goodier and Kazazian, 2008). A physiological role of LINE-1 retrotransposition in neuronal mosaicism during adult neurogenesis has also been proposed; this aspect is not discussed here and has been reviewed elsewhere (Erwin et al., 2014; Richardson et al., 2014; Faulkner and Garcia-Perez, 2017). Neverthless, LINE-1 mobilization at the level of an individual represents a threat to genome integrity. As an example, LINE-1 insertions account for 1 in every 250 pathogenic mutations in human diseases (Kazazian, 1998; Kazazian and Moran, 2017). In addition, LINE-1 unsilencing in culture can lead to numerous DSBs in the genome of human cells (Belgnaoui et al., 2006; Gasior et al., 2006), thereby decreasing cellular viability by inducing a senescence-like state (Wallace et al., 2008) or inducing apoptosis (Belgnaoui et al., 2006). ORF2p seems to nick chromosomal DNA at hundreds of different loci before each successful integration event (Gasior et al., 2006). Indeed, mutations in the EN domain of the LINE-1 ORF2 resulted in complete loss of γ-H2AX (phosphorylated histone H2A) foci, a marker of DNA damage, in HeLa cells (Gasior et al., 2006), indicating that DNA damage is mediated by the ORF2 encoded EN activity. This was also experimentally demonstrated in mouse fibroblasts (Belancio et al., 2010). Thus, all LINE-1 elements that have retained coding potential for at least the ORF2p EN are potential endogenous sources of genomic instability when unsilenced. Based on the L1Basev2 annotation (Penzkofer et al., 2017), there are about 253 individual LINE-1 elements with a complete open reading frame for ORF2p in addition to the full-length LINE-1 elements which, when activated, could constitute an additional source of genome instability in somatic cells.

**TABLE 2:** Summary of the frequency of retrotransposons and estimates about the number of open reading frames for their encoded proteins.

	LTR	LINE	SINE	References
% Human genome	9%	21%	14%	Lander et al., 2001
Integration mechanism	dsDNA + IN	TPRT + EN	TPRT + EN (from L1)	
# of potential coding loci	Predicted in^1^:12779	Predicted in^1^:21187	0	^1^ Nakagawa and Takahashi, 2016
	Predicted in^2^ ≈ 3000 Pol ORFs			^2^ Seifarth et al., 2005
# of TEs with coding potential	Predicted in^3^:42 HERVs regions with 29 Env, 13 Pol, 17 Gag ORFs	Predicted in^6^:146 flL1 with complete ORF1p and ORF2p ORFs, 107 ORF2-only	Not coding	^3^ Villesen et al., 2004
	Predicted in^5^:15 Env, 14 Pol, 25			^4^Garcia-Montojo et al., 2018, 2021
	Gag, 11 Rec, 12 Np9^4^, 8 IN			^5^ Bray et al., 2016
				^6^ Penzkofer et al., 2017
				^7^ Mills et al., 2007

*LINEs are the most represented subclass in the human genome covering 21% of the human genome with about 500,000 copies (Lander et al., 2001) of which only a small fraction of the LINE-1 family is mobile. HERVs are thought to have lost mobility, but some HERV families still encode functional proteins. LINE-1 integration is mediated by the LINE-1 encoded EN via a mechanism called targeted primed reverse transcription, TPRT. HERV elements integrate via a pre-integration complex formed by the IN protein, viral dsDNA, and host proteins. Non-autonomous SINEs do not encode protein as they use the retrotransposition machinery and form an RNP with their RNA and the LINE-1 encoded ORF1 and ORF2 for integration. Listed are predictions for the number of potential coding loci for HERV and LINE-1 elements using different sources. Of note, TEs are polymorphic in populations and these predictions are mostly based on reference genomes.*

## Multiple Layers of Line-1 Repression

Since LINE-1 activity represents a potential threat for genome integrity, several cellular factors and pathways keep these elements in check at almost every level of the LINE-1 life cycle (Figure 2). These repressive mechanisms include regulation at the epigenetic level by the binding of epigenetic modifiers to the LINE-1 sequence, at the transcriptional level by sequence-specific repressive transcription factors binding to the LINE-1 promoter in the 5′UTR (5′ untranslated region), at the post-transcriptional level by degradation mechanisms (splicing, RNA interference or RNAi, autophagy, and stress granules), at the translational level *via* RNA binding proteins, at the level of the nuclear import of the RNA, and at the integration level by several factors belonging to the DNA repair machinery (Pizarro and Cristofari, 2016). Repression at the epigenetic level is mostly accomplished by the addition of repressive histone marks, mainly trimethylation of lysine 9 of histone H3 (H3K9me3) (Bulut-Karslioglu et al., 2014; Liu et al., 2014; He et al., 2019), trimethylation of lysine 20 of histone H4 (H4K20me3) (Ren et al., 2021), and histone H1 on LINE-1 loci (Healton et al., 2020), and by DNA methylation (Hata and Sakaki, 1997; Muotri et al., 2010). This is mediated by several sequence-specific repressors that directly bind to TEs and recruit epigenetic modulators. One example of a rather general repressor of TEs is the Kruppel-associated box Zinc finger protein family (KRAB-ZFPs) which repress LTR retrotransposons (Ecco et al., 2017; Yang et al., 2017), but also LINE-1 and SVA elements (Castro-Diaz et al., 2014; Jacobs et al., 2014). KRAB-ZFPs provide a scaffold for the formation of heterochromatin on TEs. Another example for a repressive transcription factor is the homeobox protein Engrailed, which binds to the 5′UTR of LINE-1 elements and represses their expression in midbrain dopaminergic neurons (Blaudin de Thé et al., 2018). As Engrailed expression is regionally restricted (Di Nardo et al., 2018), this provides an example of region-specific TE control.

When TEs become derepressed and transcribed, the next level of repression consists of small RNAs targeted against TE transcripts. In the germline, where specific developmental states require the relaxation of epigenetic repression, the PIWI (P-element Induced WImpy testis in Drosophila)-piRNA (Piwi-interacting RNA) pathway plays an important role in TE control (Zamudio and Bourc’his, 2010). Outside of the germline, piRNA expression has been documented in somatic tissues, including the mouse brain (Lee et al., 2011), but so far the role of piRNA expression in the brain has not yet been identified. However, as a proof-of-principle, PIWIL1 (PIWI-like protein 1) overexpression from a viral vector was sufficient to repress LINE-1 induced neurodegeneration of midbrain dopaminergic neurons during oxidative stress and in the *En1*^±^ heterozygous mouse model of PD (Blaudin de Thé et al., 2018). In addition to the specific piRNA pathway, endogenous siRNAs (small interfering RNAs) suppressing TE expression have been identified in both gonadal and non-gonadal tissues. They derive from bidirectional transcription of TE-containing loci (Saito and Siomi, 2010). Other RNAi pathways, capable of degrading LINE-1 mRNA directly *via* the microprocessor complex (Drosha/DGCR8), have also been described (Heras et al., 2013, 2014). Furthermore, several cellular proteins have been identified that positively or negatively regulate LINE-1 activity through proteomic screens for cellular interactors of ORF1p or ORF2p (Pizarro and Cristofari, 2016).

Several other cellular pathways alter LINE-1 activity. Among these, autophagy is an important cellular mechanism used to degrade LTR and non-LTR retrotransposon RNA and thereby prevent retrotransposition events. Depending on the level of expression, LINE-1 mRNA localizes either to RNA granules (endogenously expressed LINE-1) or cytoplasmic stress granules (exogenous overexpressed LINE-1 RNA), but in either case, LINE-1 RNA can be degraded *via* autophagy (Guo et al., 2014). ORF1p is located in distinct foci in the cytoplasm which have been (in conditions of exogenous expression) identified as stress granules (Goodier et al., 2007, 2013). It is noteworthy that stress granules formed in the absence of an exogenous stress, indicating that ORF1p overexpression itself is recognized as a stress by the host cell. Based on their data, the authors suggest that stress granules could sequester and possibly degrade LINE-1 RNPs (ribonucleoproteins consisting of ORF1p/ORF2p and the LINE-1 mRNA) *via* P (processing)-bodies (Goodier et al., 2007), consistent with what was found for LINE-1 RNA (Guo et al., 2014). Within the stress granules, ORF1p interacts with a large number of RNA-binding proteins but interestingly, the authors also identified RNAi factors colocalizing with ORF1p, suggesting an additional layer of repression within this membraneless cytoplasmic compartment.

The proteomic screens for LINE-1 interactors also identified proteins activated through the interferon response pathway by the innate immune system. These findings are consistent with the role of the innate immune response as one of the first cellular responses activated upon viral infections (MacMicking, 2012) and the parasitic nature of LINE-1 elements. Some of these proteins colocalize with stress granules and degrade LINE-1 RNA (e.g., MOV10, Moloney leukemia virus 10 homolog). However, the exact mechanism for LINE-1 repression remains unknown for other proteins (e.g., cytidine deaminase APOBEC3) (Pizarro and Cristofari, 2016). This inflammatory response, although essential, can have pathological consequences if not regulated (Kassiotis and Stoye, 2016). Mutations in TREX1 (three prime repair exonuclease 1) or SAMHD1 (SAM domain and HD domain-containing protein 1) (Hu et al., 2015), for instance, lead to the same autoimmune disease, Aicardi-Goutières syndrome (AGS). TREX1 detects and degrades cytoplasmic ssDNA (single-stranded DNA) fragments arising from aberrant RT activity of LINE-1 or ERV (Stetson et al., 2008; Thomas et al., 2017) and possibly also released from mitochondrial damage (Sliter et al., 2018). The loss of TREX1 activity results in the accumulation of LINE-1-derived ssDNA in the cytoplasm of astrocytes, initiating (through the cGAS-STING pathway) a neurotoxic release of interferon in the extracellular medium, and leads to neurodegeneration (Thomas et al., 2017).

Finally, at the level of LINE-1 insertions or EN-mediated DSBs, the DNA repair pathway comes into play. However, while some DNA repair pathway proteins restrict LINE-1 activity (e.g., DNA excision repair protein 1, ERCC1), others such as poly(ADP-ribose) polymerase 2 (PARP2) bind to LINE-1 integration sites leading to subsequent recruitment of the replication protein A (RPA) complex to facilitate retrotransposition (for review, Pizarro and Cristofari, 2016; Miyoshi et al., 2019).

Taken together, this complex interplay of repressive mechanisms controlling TE expression ensures genomic integrity and has been shown to be of great importance in the context of neuronal cell survival (Thomas et al., 2017; Blaudin de Thé et al., 2018). However, these mechanisms seem to fail over time. We will discuss below how the aging process weakens this multi-layer cellular response (Figure 2), increasing genomic instability in the host cell and igniting (neuro)inflammation.

## Line-1 Derepression With Aging

Somatic cells express low, basal levels of LINE-1 (Faulkner et al., 2009; Belancio et al., 2010). The brain seems to provide a particularly permissive environment for LINE-1 activity (Muotri et al., 2005, 2010; Coufal et al., 2009; Macia et al., 2017) and supports higher retrotransposition rates compared to other tissues (Muotri et al., 2005; Coufal et al., 2009; Baillie et al., 2011; Evrony et al., 2012; Goodier, 2014; Suarez et al., 2018). While LINE-1 RNA expression is well documented in the human brain (Guo et al., 2018; Pereira et al., 2018; Sun et al., 2018; Savage et al., 2019), knowledge about the expression of LINE-1 encoded proteins in the brain remains scarce. Although limited by sample size, the only study, to our knowledge, using immunohistochemistry to characterize ORF1p expression in human post-mortem brain provides evidence of higher ORF1p expression levels in the brain compared to peripheral tissues and suggests that patterns of ORF1p expression might vary depending on the age of the individual, notably with regard to the subcellular localization of ORF1p (Sur et al., 2017). The mobility of TEs increases with aging in several species [*Saccharomyces cerevisiae* (Maxwell et al., 2011); *Caenorhabditis elegans* (Dennis et al., 2012); human cells in culture (De Cecco et al., 2013); mice (De Cecco et al., 2019) and mouse tissues including brain (Van Meter et al., 2014)]. As discussed above, aging is characterized by several hallmarks impacting normal cellular functions and inducing a secretory phenotype affecting surrounding cells (Di Micco et al., 2021). Aging alters repressive epigenetic modifiers, leading to heterochromatin disorganization (Misteli, 2010; Lee et al., 2020), induces autophagy inhibition (Barbosa et al., 2019) and cytoplasmic stress granule dysfunction (Cao et al., 2020), increases permeability of the nuclear membrane (Li and Lagier-Tourenne, 2018), triggers a dysfunctional innate immune response (Shaw et al., 2010) and a decline of DNA repair (Maynard et al., 2015). As shown in Figure 2, aging generally leads to a decrease in TE repressive factors (Van Meter et al., 2014), which allows TE expression. Finally, aging also favors certain environments which increase LINE-1 expression, notably oxidative stress (Rockwood et al., 2004; Blaudin de Thé et al., 2018).

The regulation of LINE-1 by SIRT6 (sirtuin 6) particularly illustrates the failure of a major LINE-1 repressive mechanism following cellular stress or aging and thus impacting cellular fitness. Indeed, during aging or in response to DNA damage, the heterochromatin-inducing protein SIRT6, a histone deacetylase, becomes depleted from LINE-1 loci, leading to derepression of LINE-1 elements (Van Meter et al., 2014). SIRT6 is a key regulator of mammalian lifespan as illustrated by the severe premature aging phenotype of Sirt6 knockout mice (Mostoslavsky et al., 2006) and the increase in lifespan of mice overexpressing Sirt6 (Kanfi et al., 2012). Interestingly, of all organs tested, the brain showed the highest upregulation of LINE-1 transcripts with aging, together with a partial loss of Sirt6 binding to the LINE-1 5′UTR. This, combined with reports of higher retrotransposition rates in the brain compared to other tissues (Coufal et al., 2009; Baillie et al., 2011) and several neurological diseases associated with a dysregulation of LINE-1 activity (Baillie et al., 2011; Jakovcevski and Akbarian, 2012), suggests that the brain might be a particularly susceptible organ to LINE-1 related aging.

Another sirtuin, Sirt7 (sirtuin 7), safeguards genome stability and cell viability (Tang et al., 2021). Among its cellular functions, Sirt7 also acts as a tethering factor, partly *via* acetylation of H3K18 (histone H3 lysine 18), between the nuclear lamina protein laminA/C and young LINE-1 sequences in mice and human cells. LINE-1 elements are thus enriched at lamin-associated domains (LADs), which are heterochromatic regions at the nuclear periphery (Zuo and Rocha, 2020), and this tethering by Sirt7 ensures LINE-1 transcriptional silencing (Vazquez et al., 2019). However, Sirt7 is downregulated during aging in hematopoietic stem cells (Mohrin et al., 2015), suggesting that this repressive mechanism of LINE-1 could be altered. Furthermore, it has been reported that aging is correlated with the decrease of LINE-1 retrotransposon promoter methylation in purified cell-free DNA from human blood (Mahmood et al., 2020).

The visionary “LINE*age*” theory has been postulated more than a decade ago (St. Laurent et al., 2010). It hypothesized that L1 acts as an “endogenous clock” which slowly erodes genomic integrity by competing with DNA break repair mechanisms and thereby negatively impacting longevity. Accumulating experimental evidence since then only confirms that LINE-1 activation might not only be an important universal hallmark of aging in various tissues, including the brain, but also a mechanistic driver of aging.

## Line-1 as a Source of Dna Damage in Neurodegeneration

Overexpression of LINE-1 in human embryonic stem cells differentiated into hippocampal neurons led to an increase in DNA DSBs (marked by γ-H2AX) which was abolished when the LINE-1 EN and RT domains were mutated (Erwin et al., 2016). The fact that LINE-1 activation can lead to DSBs in adult neurons *in vivo* and induce neurodegeneration was recently established in a mouse model of PD carrying only one allele of the homeodomain transcription factor Engrailed-1 (En1) (Blaudin de Thé et al., 2018). The homeoprotein Engrailed plays an important role in the development and survival of dopaminergic neurons during development (Simon et al., 2001; Di Nardo et al., 2007; Rekaik et al., 2015). En1 expression persists in adult midbrain dopaminergic neurons and continues to be required for the survival of these neurons. Indeed, *En1*^±^ mice lose dopaminergic neurons in the SNpc starting from 6 weeks of age (Sonnier et al., 2007). This loss is progressive and mutant mice develop PD-like motor symptoms. These mice also show early dysfunctions in nerve termini and the autophagy-lysosome pathway, reminiscent of early perturbances and retrograde degeneration patterns observed in PD. *En1*^±^ mice thus represent a valuable model for PD (Sgadò et al., 2008), used for preclinical drug testing (Ghosh et al., 2016). Further characterization of this model revealed that *En1*^±^ mice exhibit loss of repressive chromatin marks, increased LINE-1 expression, and DSBs accumulation in dopaminergic neurons in the SNpc. Importantly, it was shown that part of these DSBs result from LINE-1 activation (Blaudin de Thé et al., 2018). In line with this, DNA damage and neuronal cell death in either *En1*^±^ mice or a toxicological model of PD (direct injection of 6-hydroxydopamine in the SNpc) could be rescued by anti-LINE-1 strategies such as overexpression of PIWIL1, siRNAs targeting ORF2, or a reverse transcriptase (RT) inhibitor developed in the context of HIV/AIDS treatment, but also active against the RT enzyme encoded by ORF2p (Dai et al., 2011; Banuelos-Sanchez et al., 2019). Altogether, these studies indicate that LINE-1 activity contributes to neurodegeneration in *En1*^±^ mice and in an acute oxidative stress model of dopaminergic neurodegeneration *via* the induction of DNA damage. These studies also showed that LINE-1 retrotransposons are inducible in postmitotic dopaminergic neurons under stress conditions and stress-induced LINE-1 increase is associated with DNA damage and linked to neurodegeneration. More recently, it was shown that heterochromatin destructuration following changes in DNA methylation in dopaminergic neurons in the SNpc also resulted in LINE-1 derepression, DNA damage and neurodegeneration (Ravel-Godreuil et al., 2021a).

The active expression of TEs, especially LINE-1 retrotransposons, has now been documented in other NDs such as AD (Guo et al., 2018; Sun et al., 2018) or ALS (Pereira et al., 2018; Savage et al., 2019). Although LINE-1 retrotransposition has been reported in healthy tissues (Muotri et al., 2005, 2010; Coufal et al., 2009; Macia et al., 2017) and in the context of a large spectrum of neurological diseases (Reilly et al., 2013; Suarez et al., 2018), the question as to whether retrotransposition events take place in postmitotic neurons is still a matter of debate. In particular, the relative contribution of LINE-1 retrotransposition events in inducing genomic instability and neurodegeneration remains to be established. However, as discussed above, even in the absence of LINE-1 mobilization, the DNA strand breaks induced by LINE-1 activity can be sufficient to jeopardize genome integrity in the brain and contribute to neurodegeneration (Blaudin de Thé et al., 2018). In addition to being a source of DNA damage and genomic instability, LINE-1 could be pathogenic drivers for other pathological features of NDs. Indeed, the activity of LINE-1 loci can modulate host gene expression in various ways (Elbarbary et al., 2016; Liu et al., 2018). In addition, TE-derived proteins can be neurotoxic (Antony et al., 2004; Douville and Nath, 2017) and an increase in TE encoded proteins can be an important source of neuroinflammation. Both, gene dysregulation and neuroinflammation are common hallmarks of NDs. These aspects have recently been reviewed in depth elsewhere (Saleh et al., 2019; Tam et al., 2019a; Gorbunova et al., 2021). Further, exciting evidence suggests that some proteins linked to AD and two other neurodegenerative diseases, namely, ALS and FTD, control or interact with TEs at different levels. The protein Tau, mutated in specific forms of FTD and aggregating in several neurodegenerative diseases including AD (Orr et al., 2017) is involved in epigenetic repression of TEs (Sun et al., 2018; Ramirez et al., 2021) and TDP-43 binds TE sequences and TE transcripts (Li et al., 2012; Tam et al., 2019b).

## LINE-1, DNA Damage, and Senescence

Genomic DNA damage can also be associated with other types of cellular response, such as cellular senescence. In recent years, a role of senescent cells has been recognized in aging and aging-related pathologies (Childs et al., 2017; Kritsilis et al., 2018). It is well documented that persistent accumulation of unrepairable DNA damage in dividing cells leads to either apoptosis or senescence, characterized by permanent cell cycle arrest (d’Adda di Fagagna, 2008; Herranz and Gil, 2018) and a senescence-associated secretory phenotype (SASP). The secretion of various factors such as pro-inflammatory cytokines or matrix remodeling factors by senescent cells can promote the induction of the same phenotype in surrounding cells and drive aging and aging-related diseases. LINE-1 expression induces cellular senescence in MCF7 cells (Wallace et al., 2008), and in turn, senescence induces LINE-1 expression which activates a type-I interferon (IFN-I) response (De Cecco et al., 2019). Furthermore, several factors, such as FOXA1 (Forkhead box protein A1), TREX1, and RB1 (retinoblastoma 1), have altered expression during senescence and are also LINE-1 regulators (De Cecco et al., 2019).

While neurons cannot divide, recent evidence suggests that post-mitotic neurons could re-enter the cell cycle in the context of neurodegeneration (Nandakumar et al., 2021). Since mature neurons cannot fully terminate the cell cycle by cell division, they might rapidly exit the cell cycle and acquire a senescent-like phenotype (Jurk et al., 2012). The DNA damage resulting from the activation of LINE-1 elements could contribute to the accumulation of senescent-like neurons in various brain areas during aging and neurodegeneration. Such cells might spread this senescence-like state through SASP to neighboring neurons and thereby contribute to the chronic neuroinflammation observed in NDs. Selective ablation of senescent cells is thought to have therapeutic potential in neuroprotection (Childs et al., 2017).

## LTR Retrotransposons – Another Possible Source of Genomic Instability?

As documented above, accumulating evidence suggests that LINE-1 retrotransposons can be a source of genomic instability through the activity of the encoded EN. As DNA cleavage is an integral part of transposition, we will examine in the following section whether other TEs in the human genome could be accountable for genomic instability in specific cellular contexts. While LINE-1 are the only currently mobile TEs in the human genome, some copies of LTR retrotransposons, which comprise 8 to 9% of the human genome (Figure 1B), have retained transcriptional activity and coding potential and have been associated with human diseases, including neurodegenerative diseases. A comprehensive review on HERV pathogenicity in neurodegenerative diseases was published recently (Tam et al., 2019a).

Human LTR retrotransposons can be broadly divided into the non-autonomous MaLR and human endogenous retroviruses (HERVs), which originate from exogenous retroviruses that have infected hominoid germline cells millions of years ago (Boeke and Stoye, 1997). In contrast to endogenous retroviruses in mice, HERVs are considered non-replicating, but HERV-RNA and encoded proteins have been identified in various tissues (Garcia-Montojo et al., 2018). Although differences in structure and processing exist, ERVs follow globally the classical life cycle of retroviruses, namely, transcription from the so-called nuclear DNA provirus, a complex translation pattern of the compact RNA which includes two frameshifts, assembly of encoded proteins and RNA to envelope-mediated budding from the plasma membrane, processing of polyproteins into functional protein units by the encoded protease within the capsid, entry into a new cell, and post capsid disassembly and reverse transcription of the viral RNA into cDNA (dsDNA *via* the activity of the polymerase, Pol). Finally, a pre-integration complex containing an integrase (IN) processed from the Pol polyprotein within the capsid, “viral” dsDNA, and host proteins, enter the nucleus and, following DNA strand breaks, the viral dsDNA integrates into the host genome. Different HERV families arose through independent infections of the human germ line 10 millions of years ago (Tristem, 2000).

Very few complete HERV sequences with open reading frames for all encoded proteins have been identified in current human genomes so far (Turner et al., 2001; Belshaw et al., 2005a) as most HERV sequences have been mutated or recombined to solo-LTRs during hominoid evolution. It is clear, however, that TEs are highly polymorphic in populations (including humans) and polymorphic HERVs are linked to diseases (Wallace et al., 2018). It cannot be excluded that complete HERV sequences that are replication-competent might exist at low frequency (Belshaw et al., 2005a). The release of virus-like particles in the context of cancer has been documented (Brodsky et al., 1993; Contreras-Galindo et al., 2015), but no human HERV elements and very few mouse ERVs so far have been shown to complete the integration process. However, several loci show potential for coding for one or the other HERV protein (Table 2; Seifarth et al., 2005; Nakagawa and Takahashi, 2016), which theoretically could recombine to form a functional element or through a process combining recombination and trans-complementation (Dewannieux et al., 2006). Indeed, incomplete HERVs might retrotranspose through a process called “complementation in *trans*” (Mager and Freeman, 1995; Belshaw et al., 2005b), which requires the simultaneous expression of HERV elements encoding a polyprotein with ORFs for both Gag (group antigen) and Pol, complemented by another element (or the same), encoding a functional Env (envelop) protein. *Trans* complementation of HERVs has been experimentally demonstrated by reconstituting a HRV-K(HML-2) element, which was infectious and depended on a capsid-involving infection of another cell and could led to the reintegration of a new synthetic HERV copy *in vitro* (Dewannieux et al., 2006). Capsid-independent mobilization and amplification through retrotransposition has been suggested (Belshaw et al., 2005b), but the mechanisms by which this could be possible remain unknown. Retrotransposition in “*cis*” uses the proteins encoded by a given HERV element (or several HERVs *via trans* complementation) to “copy-and-paste” elsewhere in the genome, which requires a functional Gag and Pol, but not Env protein (as this process does not require infection). This is known for the LTR retrotransposons Ty1 and Drosophila copia (Dewannieux and Heidmann, 2005) as well as for mouse IAP elements (Ribet et al., 2008), but has not been documented for HERVs and was absent in the synthetic HERV-K *Phoenix* (Dewannieux et al., 2006). Another described mechanism for HERV amplification is the *trans*-mobilization of HERV-W elements by the LINE-1 machinery (Costas, 2002; Pavlícek et al., 2002; Grandi and Tramontano, 2017). Thus, although experimental evidence *in vivo* is lacking, HERV retrotransposition (within the same cell or *via* a capsid-involving infection of another cell) cannot be completely excluded (Romanish et al., 2010; Xue et al., 2020). Together, this theoretical framework of possible HERV retrotransposition activity implies that IN-induced genomic instability might be possible. The presence of a functional IN protein, which has been demonstrated for HERV-K (Kitamura et al., 1996) for instance, is dependent on the processing of the Pol polyprotein. However, not much is known concerning this process and whether it is possible in the absence of an infectious capsid. Some recent evidence in the context of another Pol encoded RT protein suggests that protease cleavage of the Pol polyprotein is possible in neurons and astrocytes, generating full-length RT proteins (Manghera et al., 2015) and possibly a functional IN protein. Supposing that IN is processed, it can form a so-called pre-integration complex together with the reverse transcribed dsDNA and host proteins. IN prepares the linear dsDNA ends for integration and joins these ends to the host DNA through a strand transfer reaction leaving behind DNA lesions needing to be repaired by the host DNA repair machinery (reviewed in Lesbats et al., 2016). During this process, aberrant IN activity or a failure in DNA repair (as in aging) renders the cell prone to the formation of DSB (Bray et al., 2016), which can lead to the loss of cellular functionality and, ultimately, when the cell is overwhelmed, to cell death (Hanahan and Weinberg, 2011). It remains however unclear whether this genomic instability stems from TE mobilization or the expression of the IN protein alone.

Human ERVs are expressed at low levels in the brain (Kurth and Bannert, 2010) and can be activated in several neurological diseases (Küry et al., 2018; Gröger et al., 2021). One example is ALS. HERV-K loci containing open reading frames for IN are increasingly transcribed in ALS cortical tissues (Douville et al., 2011) and RT (and Env) proteins are specifically expressed in ALS cortical neurons (Douville et al., 2011; Li et al., 2015), suggesting that IN might also be expressed. Genomic instability has been observed in ALS (Deng et al., 2014; Maizels, 2015) as well as in other neurological diseases (like schizophrenia) where HERV-K expression is upregulated (Smith et al., 2010; Kushima et al., 2017). While formal evidence of HERV-K IN-mediated DNA damage is lacking, this evidence provides a fertile ground for further studies in this direction.

## Retrotransposons as New Therapeutic Targets in Neurodegeneration

As we have developed above, the activation of coding LINE-1 and potentially HERV retrotransposons can permit the production of functional DNA cleaving proteins like the LINE-1 encoded EN or the HERV encoded IN, the presence and activity of which endanger the host genome and might contribute to aging and neurodegeneration. In the following section, we will discuss the potential of targeting retrotransposons to prevent DNA damage in the context of aging and age-related NDs.

Inhibition of retrotransposon activity can be achieved at multiple levels (Figure 2), namely, transcription, mRNA stability, translation, degradation, and enzymatic activities of the encoded proteins. Modifying environmental regulators of TEs or specific drugs intervening at various steps of the TE lifecycle are already partly available and constitute attractive tools to explore as a possible treatment of NDs.

A potentially interesting environmental regulator of RTs is caloric restriction. The impact of dietary restriction on lifespan and/or healthspan (length of life deprived of any age-related disease) in various organisms, ranging from invertebrates to non-human primates, is well documented (Fontana and Partridge, 2015). This is thought to be mediated by a downturn in the metabolic rate which, among many other effects (reviewed in Martin et al., 2007), reduces the generation of reactive oxidative species (Bianchi et al., 2016) and inflammation (Ma et al., 2020) and increases DNA repair (Rao, 2003); pathways with relevance for RT activation. Indeed, caloric restriction in mice results in a drastic decrease in the expression of TEs in liver and muscle, accompanied by an aging-related restructuration of chromatin, suggesting that dietary restriction might also modulate aging-induced chromatin remodeling and thereby reduce the transcription of TEs (De Cecco et al., 2013). Many sirtuins positively affect lifespan (Kanfi et al., 2012; Satoh et al., 2013), and are positively regulated by caloric restriction (Wątroba and Szukiewicz, 2016). The sirtuins might thus be one of the hubs for the effects of caloric restrictions linking TE regulation and aging-induced chromatin alterations.

Two sirtuins, Sirt1 and Sirt6, are of particular interest since they act as NAD^+^-dependent deacetylases of H3K9, thereby favoring the formation of H3K9me3 repressive chromatin (Zhong et al., 2010; Poulose and Raju, 2015) on LINE-1 and HERVs (Kato et al., 2018). In addition, Sirt1 activity regulates the histone methyltransferase SUV39H1 (suppressor of variegation 3-9 homolog 1) (Vaquero et al., 2004, 2007), which is also known to repress LINE-1 (Bulut-Karslioglu et al., 2014). A correlation between Sirt1 expression and LINE-1 promoter methylation levels has been identified in the human retina in the context of age-related macular degeneration (AMD) (Maugeri et al., 2019). As described above, Sirt6, in addition to being associated with aging and neurodegeneration (Kaluski et al., 2017; Portillo et al., 2021), negatively regulates LINE-1 in an age-dependent manner (Van Meter et al., 2014; Simon et al., 2019) and promotes genomic stability (Mostoslavsky et al., 2006).

Mice deficient for the DNA repair enzyme Ercc1 display an accelerated aging phenotype. Caloric restriction in these mice significantly decreases DNA damage, leads to increased lifespan and shows a protective effect on neuronal function (Vermeij et al., 2016). This is consistent with other studies showing that caloric restriction and intermittent fasting protect from cognitive decline in mouse models of AD (Halagappa et al., 2007; Stekovic et al., 2019). It is thus tempting to speculate that some of the life span-regulating effects of caloric restriction, namely, a reduction in DNA damage and inflammation, could be partly due to a decrease in RT activation upon caloric restriction *via* epigenetic containment of TEs. First evidence for a beneficial effect of caloric restriction in humans (Il’yasova et al., 2018; Redman et al., 2018) suggests that dietary interventions, including caloric restriction, could be a non-specific strategy to reduce RT expression, decrease DNA damage and inflammation, and, in turn, in the context of age-related diseases, prevent neurodegeneration.

On a transcriptional level, the homeodomain protein Engrailed could be an interesting target for LINE-1 repression in dopaminergic neurons. Engrailed is a conserved homeoprotein transcription factor involved in ventral midbrain dopaminergic neuron and cerebellum development and, in the adult ventral midbrain, specifically expressed in dopaminergic neurons and important for their survival (Alvarez-Fischer et al., 2011). Upon acute oxidative stress inflicted on midbrain dopaminergic neurons *in vivo*, Engrailed blocks cell death and restores epigenetic marks disrupted by oxidative stress (Rekaik et al., 2015). Chromatin immunoprecipitation experiments showed that Engrailed specifically binds to LINE-1 promoters and is a transcriptional repressor of LINE-1 in mice *in vivo*. By blocking LINE-1 expression, Engrailed prevented DNA damage and neuronal death induced by oxidative stress in mouse dopaminergic neurons (Blaudin de Thé et al., 2018). Importantly, the Engrailed protein can be internalized into live cells, enabling its use as a therapeutic protein (Di Nardo et al., 2018).

A more specific way to inhibit TE activity is using inhibitors of one of their encoded functional protein subunits. The one protein shared by LINE-1 and HERVs, and to which they owe their name, is the RT. Several FDA-approved drugs used in AIDS (acquired immunodeficiency syndrome) therapies that target HIV (human immunodeficiency virus) RT also efficiently inhibit the RT enzymes of LINE-1 (Dai et al., 2014; Krug et al., 2017; Liu et al., 2019) and the RT of HERVs (Garcia-Montojo et al., 2021). Blocking LINE-1 with the nucleoside reverse transcriptase inhibitor (NRTI) stavudine in the context of an acute oxidative stress, reduced DNA damage and mitigated the neurodegeneration of dopaminergic neurons (Blaudin de Thé et al., 2018). Lamivudine was shown to almost completely block the synthesis of LINE-1 cDNA, leading to a reduction in the expression of IFN-1 response genes and LINE-1 associated neuroinflammation (De Cecco et al., 2019). Similarly, inhibition of LINE-1 activity with NRTIs in Sirt-6-deficient mice (exhibiting upregulation of LINE-1, see above) rescued DNA damage (Simon et al., 2019). These results are surprising, as NRTIs are reverse-transcriptase inhibitors and do not inhibit LINE-1 EN activity. It is not clear how NRTIs can reduce EN-mediated DNA damage. It was proposed that NRTIs could terminate an integration attempt, thereby facilitating the access of the DNA damage response (DDR) machinery to damaged sites, as NRTI treatments result in less LINE-1 RNA bound to chromatin (Blaudin de Thé et al., 2018). NRTIs have been shown to be efficient in *in vitro* cell cultures or mouse models, but whether they can confer protection against neurodegeneration in diseases linked to RT activation is currently under investigation. Clinical safety and tolerability in humans have been investigated in the context of ALS in the “Lighthouse trial” (Gold et al., 2019). In this trial, a combination of NRTIs, already used in the context of AIDS, called Triumeq (abacavir, lamivudine, and dolutegravir) was tested. Triumeq was safe and well tolerated by patients and a phase 3 clinical trial has been scheduled (Gold et al., 2019). The efficacy of NRTIs to treat other NDs is currently ongoing in AD (Pizarro and Cristofari, 2016; Salloway, 2021) and continuing in ALS (National Institute of Neurological Disorders and Stroke [NINDS], 2021). Based on the current data on LINE-1 as a source of genomic instability in neurons, LINE-1 EN inhibitors would be of interest but are unfortunately not available.

In addition to RT inhibitors, efficient inhibitors of HIV IN were developed to treat AIDS. Although with lower efficacy, these inhibitors are also effective against the HERV-K/HML-2 IN *in vitro* (Contreras-Galindo et al., 2017; Tyagi et al., 2017). As discussed above, based on the scarcity of evidence for HERV-mediated genomic instability, the use of IN inhibitors is of less interest in the context of NDs.

## Conclusion

**FIGURE 3:**
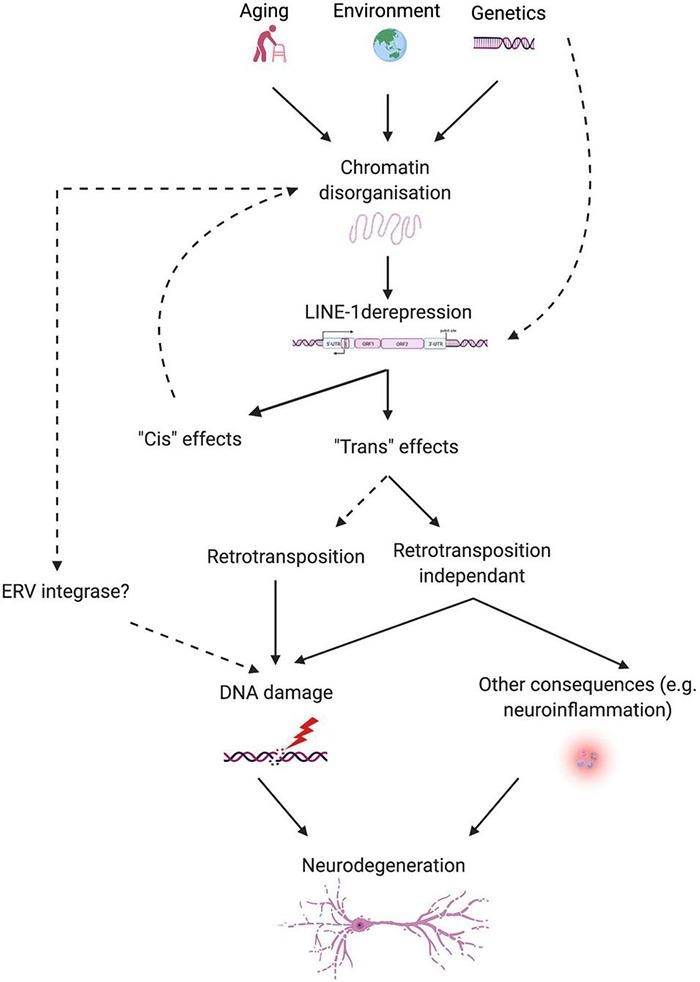
Schematic overview of the proposed link between aging, LINE-1 elements, and neurodegeneration. Aging, environment, and possibly genetic predisposition lead to chromatin disorganization, which releases epigenetic repression on LINE-1 elements. Some proteins relevant for neurodegenerative diseases (i.e., Tau and TDP-43) control TEs, including LINE-1, at several levels. LINE-1 transcriptional activation can lead to “*cis*” and “*trans*” effects. “*Cis*” effects concern local effects on chromatin organization or gene expression. Intronic LINE-1 might lead to local gene dysregulation by protein truncations *via* premature polyA signaling, alterations in splicing of the hosting gene, generation of antisense transcripts, *etc.*, or, in the case of intergenic LINE-1, to the demasking of enhancers, transcription factor binding sites, changes in the 3D organization of the chromatin, *etc*. “*Trans*” effects can be mediated by actual retrotransposition events or retrotransposition-independent consequences of LINE-1 activation like DNA damage and neuroinflammation. Other sources of DNA damage can add onto this, stemming from environmental influences, the aging process, neurodegenerative processes, and possibly other TEs, like ERVs, resulting in neurodegeneration.

In view of the evidence discussed in this review, a novel pathogenic axis for NDs emerges, linking age and neurodegeneration based on the aging-induced alteration of heterochromatin organization, subsequent LINE-1 derepression, and LINE-1-related genomic instability leading to neurodegeneration. This hypothesis reconciles the transposon theory (Driver and McKechnie, 1992), extended by the LINEage theory stipulating the importance of non-retrotransposition dependent DNA damage in the aging process (St. Laurent et al., 2010), with the heterochromatin theory of aging (Villeponteau, 1997) and provides a molecular basis to explain why age is the major risk factor for NDs. Derepression of transposable elements including LINE-1 can have multiple consequences on cellular functions of which genomic instability appears to be of major importance. Examples of neurodegenerative processes favoring TE derepression are emerging (Guo et al., 2018; Sun et al., 2018) and suggest that genetic risk together with environmental factors and age in combination might determine the individual risk to develop a neurodegenerative disease (Figure 3). As such, this pathogenic axis could be shared by many age-related NDs and regional susceptibility could be determined by the combination of TE regulatory factors specific for certain neuronal populations or brain regions. Of specific interest is the potential of this emerging axis not only to foster the understanding of the pathogenesis of NDs but also to provide a point of attack for the development of urgently needed disease-modifying treatments. Much is still to learn and the coming years will likely increase our understanding of the role that TEs play in the physiology and pathophysiology of the brain.

## Author Contributions

All authors listed have made a substantial, direct, and intellectual contribution to the work, and approved it for publication.

## Conflict of Interest

The authors declare that the research was conducted in the absence of any commercial or financial relationships that could be construed as a potential conflict of interest.

## Publisher’s Note

All claims expressed in this article are solely those of the authors and do not necessarily represent those of their affiliated organizations, or those of the publisher, the editors and the reviewers. Any product that may be evaluated in this article, or claim that may be made by its manufacturer, is not guaranteed or endorsed by the publisher.

## Abbreviations

AD: Alzheimer’s disease
DSBs: DNA double-strand breaks
EN: endonuclease
En1: Engrailed-1
ERV: endogenous retrovirus
HERV: human ERV
IN: integrase
LINE-1: long interspersed element-1
NDs: neurodegenerative diseases
NRTI: nucleoside reverse transcriptase inhibitor
PD: Parkinson’s disease
RT: reverse transcriptase
SNpc: *substantia nigra pars compacta*
TEs: transposable elements.
